# Supplementary material for: Effect of health education in the control of schistosomiasis in Dass Emirate Council of Bauchi State, Nigeria: An intervention study
Source: PLoS Negl Trop Dis. 2025 Jan 9;19(1):e0012358. doi: 10.1371/journal.pntd.0012358 (PMC11753707; doi:10.1371/journal.pntd.0012358)
Supplement: Attachment — Submitted filename: Review report PNTD-D-24-00915.pdf [file pntd.0012358.s001.pdf]

Review report PNTD-D-24-00915

Title: Effect of health education in the control of schistosomiasis in Dass Emirate council of Bauchi state, Nigeria: an intervention study

The abstract is well-structured, summarizing the objective, methods, results, and key findings. However, it could benefit from a more detailed explanation of the intervention's components. The significance of the findings is appropriately highlighted, particularly the reduction in schistosomiasis prevalence and the improvement in knowledge post-intervention.

The authors provide a thorough overview of schistosomiasis, emphasizing its public health importance, particularly in sub-Saharan Africa. The literature review is relevant and highlights the need for integrated approaches, including health education, to combat the disease. However, the introduction could benefit from a clearer articulation of the study's novelty and how it addresses gaps in the existing literature. The study's objective is clearly stated, aiming to assess the impact of health education on the prevalence of schistosomiasis and the knowledge, attitudes, and practices (KAP) of the community. This objective is well-aligned with the study design and expected outcomes.

The method and the procedure use to address the aims are acceptable.

The pre-intervention data reveals a high prevalence of schistosomiasis and generally low levels of knowledge and preventive practices, which aligns with the study’s context. The manuscript effectively highlights the significant improvements in knowledge and reductions in schistosomiasis prevalence post-intervention.

The discussion appropriately interprets the findings in the context of existing literature, particularly the significant reduction in schistosomiasis prevalence and the increase in community knowledge. The manuscript does well to link these outcomes to the health education intervention.

The recommendation for integrating health education into broader schistosomiasis control programs is particularly appreciated and the conclusion effectively summarizes the study's key findings and their implications. It is concise and ties back to the study's objectives.

Overall appreciations

The manuscript presents a well-conducted intervention study with clear and significant findings regarding the impact of health education on schistosomiasis control in a high-prevalence area. The strengths of the study include its relevance to public health, the use of a quasi-experimental design, and the significant improvements observed in community knowledge and disease prevalence. However, the manuscript could benefit from additional details on the intervention itself, a more thorough discussion of the findings, and a more

robust consideration of the study’s limitations. Overall, the study makes a valuable contribution to the field of parasitic disease control and provides actionable insights for public health practitioners. Additionally, the manuscript and the study present some weaknesses that need to be clearly addressed by the authors.

Study area:

Authors should provide map ma of the study area (Dass Emirate), located in Nigeria map and in the Bauchi state map

Study Design and Sampling:

- The Authors utilizes a quasi-experimental design, which is weaker compared to randomized controlled trials. Without randomization, there's a higher risk of selection bias, making it difficult to attribute changes solely to the intervention.

- The study population is described as residents of Dass Emirate Council, but there is insufficient detail about the demographic diversity of the sample. This lack of detailed demographic information makes it challenging to generalize the findings to other populations.

- The authors mention the use of a multistage sampling technique but does not provide enough details about the sampling stages, how participants were selected, or how the sample size was determined using Fisher's formula.

- **Intervention**: the manuscript could provide more details on the content, delivery methods, and duration of the intervention. Understanding the specific educational materials and strategies used would enhance the reproducibility of the study.
-

Data Analysis:

The authors primarily use chi-square tests for comparing pre- and post-intervention data. While chi-square tests are appropriate for categorical data, other statistical methods, such as regression analysis, could have been employed to control for potential confounders. There is a lack of discussion regarding potential confounders or biases that might have influenced the study outcomes. For instance, changes in environmental conditions, access to healthcare, or concurrent public health campaigns could have impacted the results.

Intervention Details:

The health education intervention is not clearly described. The Authors mention the use of a training manual but provide minimal information on the content, duration, or methods used in the intervention. More detailed information on the educational content, delivery

methods, and duration would be beneficial for replicability and understanding the effectiveness of the intervention.

The manuscript does not mention any follow-up period to assess the long-term impact of the health education intervention. A follow-up study would help determine whether the observed changes in knowledge, attitudes, and practices are sustained over time. This could be one of the limitations and/or future direction

Results Interpretation:

The manuscript reports that the reduction in schistosomiasis prevalence was statistically significant, but it does not address the clinical significance of this finding. A discussion on whether the observed reduction is meaningful in terms of public health impact is lacking.

The manuscript mentions that improvements in knowledge, attitudes, and practices were not statistically significant in some cases, but it does not explore why this might be the case or what could be done to enhance the effectiveness of the intervention.

Some statements, particularly in the discussion, lack citation or rely on outdated sources. The manuscript could benefit from more current references to support its claims.

Authors should provide information about age, sex and integrate these parameters in their analysis. That will give more details about the strategies for policies makers and empower the quality of data of this manuscript.

Discussion

The manuscript does well to link these outcomes to the health education intervention, although it could delve deeper into the mechanisms by which education influenced these changes.

While the manuscript touches on the limitations related to the quasi-experimental design and self-reported data, it could expand on other potential limitations, such as the generalizability of the findings to other regions or the long-term sustainability of the intervention’s impact.

The authors could provide more concrete suggestions for future research, such as exploring different educational approaches or assessing long-term outcomes.

Here are some writing mistakes that need to be corrected

In the Abstract section

Sentence: "Schistosomiasis has been recognized by WHO as major contributor to mortality and morbidity particularly in Sub Sahara Africa where it is mostly prevalent."

Correction: "Schistosomiasis has been recognized by WHO as a major contributor to mortality and morbidity, particularly in Sub-Saharan Africa, where it is most prevalent."

In the Introduction section

Sentence: "Schistosomiasis, commonly known as bilharzia, is a parasitic disease caused by trematode flatworms of Schistosoma."

Correction: "Schistosomiasis, commonly known as bilharzia, is a parasitic disease caused by trematode flatworms of the genus *Schistosoma*."

In the Methods section

Sentence: "The data collected were analyzed with SPSS software package version 25 and p - value less than 0.05 was taken as statistically significant."

Correction: "The data collected were analyzed using SPSS software version 25, and a p-value of less than 0.05 was considered statistically significant."

In the Results section

Sentence: "More than half, (207, 59.1%) reported that someone in their community has been diagnosed with Schistosomiasis."

Correction: "More than half, (207, 59.1%) reported that someone in their community had been diagnosed with schistosomiasis."

Sentence: "Knowledge of schistosomiasis increased to 87.0% at post-intervention and this was statistically significant at $p < 0.001$."

Correction: "Knowledge of schistosomiasis increased to 87.0% post-intervention, which was statistically significant at $p < 0.001$."

In the Discussion section

Sentence: "This reveals significant shifts in the community's perceptions and reported experiences with the disease."

Correction: "This reveals significant shifts in the community’s perceptions and experiences with the disease."

General Comments:

- **Consistency in Terminology:** There are inconsistencies in the capitalization of "schistosomiasis." It should be consistently lowercase unless it is the first word in a sentence.
- **Punctuation:** Ensure proper use of commas, especially in complex sentences, to improve readability.
- **Subject-Verb Agreement:** Double-check for subject-verb agreement throughout the manuscript.
