# Supplementary material for: Effect of health education in the control of schistosomiasis in Dass Emirate Council of Bauchi State, Nigeria: An intervention study
Source: PLoS Negl Trop Dis. 2025 Jan 9;19(1):e0012358. doi: 10.1371/journal.pntd.0012358 (PMC11753707; doi:10.1371/journal.pntd.0012358)
Supplement: Attachment — Submitted filename: RESPONSE TO REVIEWERS.docx.pdf [file pntd.0012358.s003.pdf]

Title: Effect of Health education in the control of Schistosomiasis in Dass Emirate council of Bauchi State: An intervention study

Authors: Sunday Charles Adeyemo, Gbadebo Jimoh Oyedeji, James Atolagbe, Oladunni Opeyemi, Sunday Olanrewaju, Calistus Akinleye, Funsho Olagunju, Eniola Dorcas Olabode, Abdulwaris Salisu Maleka

Manuscript ID: PNTD-D-24-00915

Dear Editor and Reviewers,

On behalf of my co-authors, I extend our deep appreciation for the opportunity to make corrections to our manuscript. Thank you for the time and effort you have invested in providing detailed feedback on our work. Your comments and suggestions are of great value and have significantly enhanced the quality of our manuscript.

The reviewer's comments have been analyzed carefully and we have made effort to address them in our revisions. The following is a comprehensive list of our responses and the corresponding revisions, which we have effected.

We have taken great care to ensure that our manuscript now meets the high standards expected by PLoS One and hope that it will be found acceptable for publication. We are once again thankful for the diligent work of the editorial team and the reviewers.

Best regards

Yours Sincerely

Dr Sunday Charles Adeyemo

Please find the following Response to the comments of reviewers:

Reviewer 1

Comment 1: Somewhat, no sufficient details were given about the intervention.

Response: Thank you for bringing this to our attention, we apologize for not supplying sufficient information about the intervention. The intervention involved structured health education programs and campaigns in the communities. The health education was conducted by trained health educators for four hours per contact in all the ten communities selected in Dass Emirate. The training targeted population at risk and major stakeholders including school pupils, market people, religious leaders, farmers and herders in 10 communities of Dass Emirate of Bauchi where schistosomiasis was mostly prevalent. The duration of the health education was 4 weeks due to the nature of occupation of the respondents who were predominantly farmers and fisher men who needed to go to their various farms and the insecurity issues peculiar with the study location. The training manual of the Federal Ministry of Health of Nigeria which contained information on the identification of schistosomiasis symptoms, the mode of transmission as well as preventive measures of schistosomiasis was adapted during the health education. The health educators ensured the health education sessions were interactive in order to understand the perception and attitude of the community members towards schistosomiasis. They further emphasized the need for a change in behavior in order to promote the prevention of schistosomiasis. The strategies of the health education campaign involved community participation by creating a schistosomiasis booth at the community field. Another strategy was peer education which involves identification of champions of the group which included community leaders, religious leaders, district heads, youth leaders, market women and leaders and principals of Almanjiri schools who served as peer educators to increase awareness about the prevention and control of schistosomiasis in the communities. Also, community outreach was done to hard-to-reach places.

Comment 2: Thank you for the suggestions for the minor revisions. All the revisions have been effected in the new submission.

Comment 3: The plan for the training should be given e.g. contents, duration, staffing, attendance and estimated costs and comment on the feasibility of application in other endemic areas. This is a major revision.

Response: We are sorry for not including this important information in our submission. It has been added in the new submission. A total of 17 Research Assistants who served as enumerators and health educators were recruited. The research assistants were dwellers of the communities who can speak and understand Hausa language. This is to ensure proper communication between them and the community members. 15 of the research assistants were health care workers with the primary health care board in Bauchi State, who helped in engaging the community members at the community health centers. Two were students of Bauchi State University studying Statistics. The head of the healthcare workers have Masters in Health Care Economics while others have national diploma (ND) and higher national diploma (HND) in Health education, Health information technology and community health from School of Health. This aided adequate understanding of the research work and communication between them and the researcher, especially during the training. A total of 345 respondents were in attendance out of 350 that were recruited pre-intervention. An estimated cost of 2million naira ($1250) was spent on the study. The intervention is applicable in other endemic areas.

Reviewer 2

Comment 1: The quality of the figure need to be improved.

Response: Thank you for bringing this to our notice. We have improved the quality of the figures.

Comment 2: The manuscript could benefit from additional details on the intervention itself, a more thorough discussion of the findings, more robust consideration of the study's limitation.

Response: Thank you for your encouraging comments. We are sorry for any hidden information in the manuscript. A detailed description of the intervention has been included in the new submission.

Reviewer 3

Comment 1: The Statistical Analysis needs major revision and clarity.

Response: We are sorry for the ambiguity in the statistical analysis. The data collected were analyzed with SPSS software package version 25, and a p- value of less than 0.05 was considered statistically significant. Descriptive statistics were done for all variables. Comparison of variables

at pre-and post-intervention was done using McNemar’s test as a test of association. Regression analysis was used to identify predictors of Knowledge, Attitude and Practices (KAP) scores.

Additional comments

Comment 1: The Authors utilizes a quasi-experimental design, which is weaker compared to randomized controlled trials. Without randomization, there's a higher risk of selection bias, making it difficult to attribute changes solely to the intervention.

Response: We appreciate your feedback on this, however, we are of the opinion that, since we are not testing a new drug, quasi-experimental design remains the suitable method considering the objectives and scope of the study. In the context of the research, randomization is not feasible. Also, causal association is not part of the scope of the study, hence, it is not necessary to use clinical trial study design. To minimize the effect of cofounders and bias, the selection of respondents was a multi-stage sampling technique which involved stratification.

Comment 2: The manuscript does not mention any follow-up period to assess the long-term impact of the health education intervention. A follow-up study would help determine whether the observed changes in knowledge, attitudes, and practices are sustained over time. This could be one of the limitations and/or future direction

Response: Thank you for bringing this to our notice. A follow up study would not have been out of place, but it is practically impossible in this case because Bauchi state is one of the North Eastern states that have been plagued with insecurity due to activities from Boko Haram and banditry activities for more than a decade now. It is quite challenging to carry out a follow-up study.

Comment 3: The manuscript reports that the reduction in schistosomiasis prevalence was statistically significant, but it does not address the clinical significance of this finding. A discussion on whether the observed reduction is meaningful in terms of public health impact is lacking.

Response: Thank you for this comment. The reduction is of public health relevance as it serves as an important measure in combating schistosomiasis in other endemic areas. This has been included in the new submission.

Comment 4: The manuscript mentions that improvements in knowledge, attitudes, and practices were not statistically significant in some cases, but it does not explore why this might be the case or what could be done to enhance the effectiveness of the intervention.

Response: We appreciate your feedback on this. The lack of statistically significant difference in some cases might have been due to the short duration of the health education intervention program due to insecurity issues in the study location. An intervention done for longer duration would be recommended, even though it was not feasible in our own case, so as to avoid loss to attrition and also because of the security situation.

Comment 5: Some statements, particularly in the discussion, lack citation or rely on outdated sources. The manuscript could benefit from more current references to support its claims.

Response: Thank you for bringing this to our notice. More recent citations have been added to the manuscript.

Comment 6: Authors should provide information about age, sex and integrate these parameters in their analysis. That will give more details about the strategies for policies makers and empower the quality of data of this manuscript.

Response: We are sorry for omitting this in the initial submission. Detailed information about the sociodemographic characteristics of the respondents has been included in the new submission.

Comment 7: While the manuscript touches on the limitations related to the quasi-experimental design and self-reported data, it could expand on other potential limitations, such as the generalizability of the findings to other regions or the long-term sustainability of the intervention's impact.

Response: Thank you for your feedback on this. Other potential limitation is that the prevalence of schistosomiasis was not clinically confirmed which could have impact on generalizability of the study findings.

Comment 8: The authors could provide more concrete suggestions for future research, such as exploring different educational approaches or assessing long-term outcomes.

Response: Thank you for bringing this to our notice. We further suggested that future research should focus on conducting long-term follow-up studies to assess the sustained impact of health education interventions. Evaluating changes in knowledge, attitudes, and practices over an extended period can provide insights into the durability of behavior change.
