# Supplementary material for: Effect of health education in the control of schistosomiasis in Dass Emirate Council of Bauchi State, Nigeria: An intervention study
Source: PLoS Negl Trop Dis. 2025 Jan 9;19(1):e0012358. doi: 10.1371/journal.pntd.0012358 (PMC11753707; doi:10.1371/journal.pntd.0012358)
Supplement: Attachment — Submitted filename: RESPONSE_TO_REVIEWERS.docx [file pntd.0012358.s004.docx]

Title: Effect of Health education in the control of Schistosomiasis in Dass Emirate council of Bauchi State: An intervention study

Authors: Sunday Charles Adeyemo, [Gbadebo Jimoh Oyedeji,](https://pubmed.ncbi.nlm.nih.gov/?term=Oyedeji+GJ&cauthor_id=32101120) James Atolagbe, Oladunni Opeyemi, Sunday Olanrewaju, Calistus Akinleye, Funso Olagunju, Eniola Dorcas Olabode, Abdulwaris Salisu Maleka

Manuscript ID: PNTD-D-24-00915

Dear Editor and Reviewers,

On behalf of my co-authors, I extend our deep appreciation for considering our manuscript and giving us the opportunity to make corrections. Thank you for the time and effort you have invested in providing detailed feedback on our work. Your comments and suggestions are of great value and have significantly enhanced the quality of our manuscript.

The reviewer’s comments have been analyzed carefully and we have made effort to address them in our revisions. The following is a comprehensive list of our responses and the corresponding revisions, which we have effected.

We have taken great care to ensure that our manuscript now meets the high standards expected by PLoS One and hope that it will be found acceptable for publication. We are once again thankful for the diligent work of the editorial team and the reviewers.

Best regards

Yours Sincerely

Dr Sunday Charles Adeyemo

Please find the following responses to the comments of reviewers:

**Reviewer 1**

Comment 1: In editing the manuscript, please note that "schistosomiasis" is the name of the disease and should not be capitalized like the taxonomic name of the parasite.

Response: The word “schistosomiasis” has been changed to lower case except when it is at the beginning of a sentence.

**Reviewer 3**

Comment 1: The structure is generally clear, but the flow could be improved. The description of the sampling techniques and intervention processes is detailed but occasionally overwhelming due to the amount of technical information provided in a single paragraph. Breaking down the sampling method into distinct sections (e.g., Stage 1, Stage 2, Stage 3) with subheadings could improve readability.

Response: We are sorry for any inconveniences caused. The paragraphs have been broken into more paragraphs to enhance readability. The sampling method has been broken into the different stages.

Comment 2: Bias and Limitations: The study does not discuss potential sources of bias (e.g., selection bias, response bias) or limitations related to its quasi-experimental design. Without a control group, it’s difficult to rule out external factors that may have influenced the post-intervention results. This could have been acknowledged and addressed in the methodology section.

Response: We agree that quasi-experiment has its own weaknesses compared to randomized control trial as a design. However, we are of the opinion that the research still achieved the objectives of the study, as establishing causal association was not part of the study objectives, and the study does not involve clinical trial of a new drug or a cohort study.

**Reviewer 4**

Comment 1:  I would change the colours of the table to a more neutral colour. Please consider changing the graph colours to a clearer and user friendly colour.

Response: The colour of figure 1 has been changed to enhance readability.
